# The difference in the composition of gut microbiota is greater among bats of different phylogenies than among those with different dietary habits

**DOI:** 10.3389/fmicb.2023.1207482

**Published:** 2023-07-28

**Authors:** Min Guo, Siwei Xie, Junhua Wang, Yuzhi Zhang, Xiangyang He, Pengfei Luo, Jin Deng, Chunhui Zhou, Jiao Qin, Chen Huang, Libiao Zhang

**Affiliations:** ^1^Guangdong Key Laboratory of Animal Conservation and Resource Utilization, Guangdong Public Laboratory of Wild Animal Conservation and Utilization, Institute of Zoology, Guangdong Academy of Sciences, Guangzhou, China; ^2^College of Mathematics and Informatics, South China Agricultural University, Guangzhou, China; ^3^College of Life Sciences, South China Normal University, Guangzhou, China; ^4^Institute of Plant Protection, Guangdong Academy of Agricultural Sciences, Guangzhou, China; ^5^Dr. Neher’s Biophysics Laboratory for Innovative Drug Discovery, State Key Laboratory of Quality Research in Chinese Medicine, Macau University of Science and Technology, Taipa, Macao SAR, China

**Keywords:** bat, phylogeny, dietary habit, gut microbiota, dietary shift

## Abstract

Bats have a very long evolutionary history and are highly differentiated in their physiological functions. Results of recent studies suggest effects of some host factors (e.g., phylogeny and dietary habit) on their gut microbiota. In this study, we examined the gut microbial compositions of 18 different species of bats. Results showed that Firmicutes, Gammaproteobacteria, and Actinobacteria were dominant in all fecal samples of bats. However, the difference in the diversity of gut microbiota among bats of different phylogenies was notable (*p* = 0.06). Various species of Firmicutes, Actinobacteria, and Gammaproteobacteria were found to contribute to the majority of variations in gut microbiota of all bats examined, and *Aeromonas* species were much more abundant in bats that feed on both insects and fish than in those of insectivores. The abundance of various species of *Clostridium*, *Euryarchaeota*, and ancient bacterial phyla was found to vary among bats of different phylogenies, and various species of *Vibrio* varied significantly among bats with different dietary habits. No significant difference in the number of genes involved in various metabolic pathways was detected among bats of different phylogenies, but the abundance of genes involved in 5 metabolic pathways, including transcription; replication, recombination, and repair; amino acid transport and metabolism; and signal transduction mechanisms, was different among bats with different dietary habits. The abundance of genes in 3 metabolic pathways, including those involved in stilbenoid, diarylheptanoid, and gingerol biosynthesis, was found to be different between insectivorous bats and bats that feed on both insects and fish. Results of this study suggest a weak association between dietary habit and gut microbiota in most bats but a notable difference in gut microbiota among bats of different phylogenies.

## Introduction

Gut microbiota is composed of many bacteria, fungi, viruses, and other organisms in the gastrointestinal system. The composition of gut microbiota in animals varies greatly in their early life but becomes stable in adulthood (Lim et al., 2015; Yin et al., 2020). Many large-scale studies have revealed effects of gut microbiota on the well-being of the host (Wan et al., 2020; Kuang et al., 2022). Host factors such as phylogeny, physiology, behavior, and dietary habit have also been shown to affect the composition of gut microbiota (Benson et al., 2010; Kurilshikov et al., 2021).

A recent large-scale study of the gut bacteria of bats, amphibians, and birds showed that bats and birds share similar profiles of gut bacteria (Song et al., 2020), suggesting that an innate ability, such as flying, may have similar effects on gut bacteria in animals of different phylogenies. In contrast, other studies disclosed a link between specific microorganisms and their hosts (Brucker and Bordenstein, 2012; Easson and Thacker, 2014; Sanders et al., 2014; Brooks et al., 2016). Such relationship is referred to as phylosymbiosis that predicts host clades harbor distinguishable microbial communities (Brucker and Bordenstein, 2012) and is common in some animals, such as sponges (Easson and Thacker, 2014), Nasonia (Brucker and Bordenstein, 2012), ants, and apes (Sanders et al., 2014). These findings suggest a relationship between the composition of gut microbiota and host evolutionary history, behaviors, and ecological factors (Zepeda Mendoza et al., 2018; Lutz et al., 2019).

Bat is the second largest order of mammals and have a long evolutionary history, distinct physiology, diverse dietary habits, echolocations, and flying ability (Adams and Pedersen, 2013). Similar to carnivores, the gut microbiota of bats are dominated by Firmicutes and Proteobacteria (Sun et al., 2020). It has been shown that the compositions of gut bacteria of bats vary with their diet, sex, age, mass, geographic location, physiology, reproduction, and symbiotic parasites (Phillips et al., 2012; Carrillo-Araujo et al., 2015; Lutz et al., 2019). Although the effects of host factors on gut bacteria have been described (Phillips et al., 2012; Carrillo-Araujo et al., 2015; Lutz et al., 2019), the mechanisms of such effects are unknown. We hypothesized that host phylogeny and dietary habits could affect the diversity of gut microbiota and therefore conducted this study to compare the compositions of gut microbiota in bats of different phylogenies and those of insect-eating (insectivores) bats, fruit-eating (frugivores) bat, and bats that feed on both insects and fish, aiming to uncover the impact of various host factors on the gut microbiota of bats.

## Materials and methods

### Samples

A total of 24 bats of 18 different species belonging to 8 genera and 3 families (Pteropodidae, Rhinolophidae, and Vespertilionidae) were captured from Yanyan Cave, Huizhou City, China. These bats included 6 *Monascus pilosus* bats, 2 *Monascus chinensis* bats, and one each of the following: *Monascus altarium*, *Monascus davidii*, *Monascus longipes*, *Rhinolophus pearsonii*, *Rhinolophus pusillus*, *Rhinolophus siamensis*, *Rhinolophus affinis*, *Rhinolophus macrotis*, *Hypsugo pulveratus*, *Eptesicus serotinus*, *Pipistrellus abramus*, *Murina huttoni*, *Murina aurata*, *Miniopterus pusillus*, *Macropus fuliginosus*, *Rousettus leschenaultii* (Wilson and Mittermeier, 2019). These bat species were selected because they have different dietary habits in addition to different phylogenies. Among the 18 bat species, *M. pilosus* bats feed on both insects and fish, *R. leschenaultii* bat is frugivores, and the remaining bats are insectivores (Wilson and Mittermeier, 2019).

To collect fecal samples, each bat was kept in a clean cloth bag for about 5 h. Fecal particles were then collected and placed in 2 mL tubes. For those with no fecal particles, anal swabs were used. A total of 24 fecal samples were collected (one sample from each bat). The samples were immediately snap frozen in liquid nitrogen and then stored in a −80°C freezer until used.

### DNA extraction, metagenomic assembly, and taxonomical annotation

DNA was extracted from fecal samples using the QIAamp^®^ Fast DNA Stool Mini kit following manufacturer’s instructions without any modifications. DNA samples were then subjected metagenomic analysis by next generation sequencing (NGS) with a NovaSeq 6000 sequencer (read length: 150 bases; sequencing depth: 10 Gb; paired-end sequencing by Shanghai Origingene Corporation, China).

To filter out low-quality and adapter contaminated reads, the raw reads were subjected to quality control by SOAPnuke (1.5.6; Chen et al., 2018). The high-quality reads were assembled by Megahit^1^ (Li et al., 2015). MetaGeneMark^2^ (Zhu et al., 2010) was employed to search for coding sequences (CDSs) in assembled contigs longer than 100 bp. To construct a non-redundant gene set, genes of all samples were merged and clustered with 95% identity by Cd-hit (v4.6.4)^3^ (Li and Godzik, 2006). Taxonomic identification of genes in the non-redundant gene set was performed with Blastp (v2.2.28)^4^ (Camacho et al., 2009) by aligning the genes against those in the Nucleotide Sequence Database (Nr database; v20170519) at an *e*-value ≤1e−5. When the gene length coverage was less than 50%, the gene was assigned an “Unclassified” status. Genes were annotated by aligning high quality reads to the non-redundant gene set using Bowtie2 (v2.2.5; Langmead and Salzberg, 2012), and gene abundance was calculated by PathoScope2 (v2.0.6; Hong et al., 2014). Species abundance was determined by the sum of assigned gene counts. The taxonomic tree of microbes was built according to the NCBI taxonomy. Divergence time of bat species in the phylogenetic tree was determined by TimeTree 5^5^ (Kumar et al., 2022).

### Gene function and metabolic pathway annotations

The non-redundant gene set was annotated by blasting translated proteins against the database of Clusters of Orthologous Groups of proteins (COG) (v20090331) and that of Kyoto Encyclopedia of Genes and Genomes (KEGG) (v81). COG-annotated gene functions were visualized using the boxplot function of the Lattice package in the R software. Significantly changed gene functions and pathways between groups were detected by the Wilcoxon test of the R software (adjusted *p* < 0.05, by the method of Holm).

### Evaluation of microbial diversity

To assess the compositional homogeneity of the microbial communities in samples, Shannon–Wiener index (Shannon, 2001) was calculated using vegan, ggplot2, RColorBrewer, and reshape2 packages of the R software. Weighted principal component analysis (PCA) was executed using ggbiplot, ggrepel, and ggplot2 packages. *Z*-score normalization (Shalabi et al., 2006) was performed with the prcomp package prior to calculation of the principal components. The ggbiplot package was used to visualize the distribution of each sample in PC1 and PC2. Significant difference between groups was determined using the adonis package of the R software (permutations = 999, by the method of Euclidean; Anderson, 2001).

### Identification of significant gut microorganisms and host metabolic pathways

To identify the significant gut microorganisms, 500 tree models were constructed by random forest (RF) modeling. Each tree model was trained by bootstrapping samples from all samples; those samples unemployed in the training (~36.8%) were used to test the accuracy of the tree model (Breiman, 2001). RF models were built by the package randomForest of the R software (with parameters: ntree = 500, mtry = sqrt(*p*) where *p* is number of variables, replace = TRUE, maxnodes = NULL, oob.prox = proximity, nPerm = 1, maxnodes = NULL) to analyze data of bats with different dietary habits and those of different phylogenies by random permutation of species-level abundance profiles. The out-of-bag (OOB) error rate was used to assess the accuracy of modeling. Microbial influence value was determined by the mean decrease in the Gini index of each node of the constructed model, and a higher value indicated a more significant effect on the composition of gut microbiota. The number of microbial species was determined by repeating RF modeling 5 times with 10-fold cross validation.

The significantly changed (*p* < 0.01) abundance of bacterial species between insectivorous bats and bats that eat both insects and fish were determined by Metastats non-parametric multiple tests with *p*-values adjusted for false discovery rate (FDR) to aviod errors (White et al., 2009). The significant difference in the number of genes involved in certain functions or pathways between groups were detected by the Wilcoxon test (adjusted *p* < 0.05 by the method of Holm). The abundance profiles of various significant microbes were visualized in heat map by packages gplots, fields, and akima of the R software.

## Results

### Sequencing data

**Figure 1:**
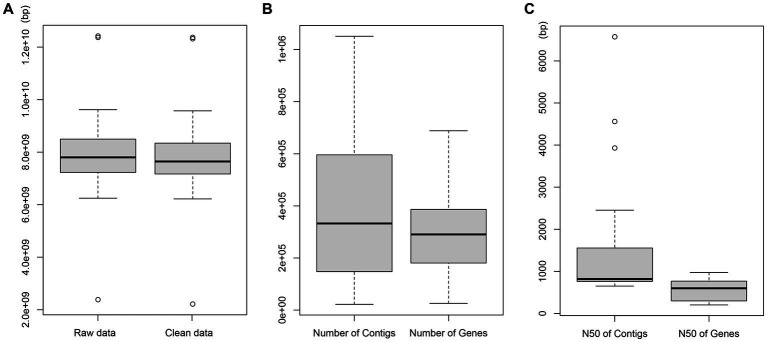
Sequencing data. **(A)** Length distribution of raw data. **(B)** Number distribution of contigs and genes. **(C)** Length distribution of N50 contigs and genes.

The NGS performed with the NovaSeq 6000 platform on the 24 bat fecal samples generated approximately 7.9 GB raw data per sample. After cleaning, an average of 7.8 Gb clean data were obtained from each sample with 22,213–1,050,926 contigs and 25,969–688,291 genes. The average N50 of all genes was 570 bp (Figure 1), and 7,525,231 unique genes were obtained from all samples.

### Variations in gut microbiota among bats of different phylogenies or with different diets

**Figure 2:**
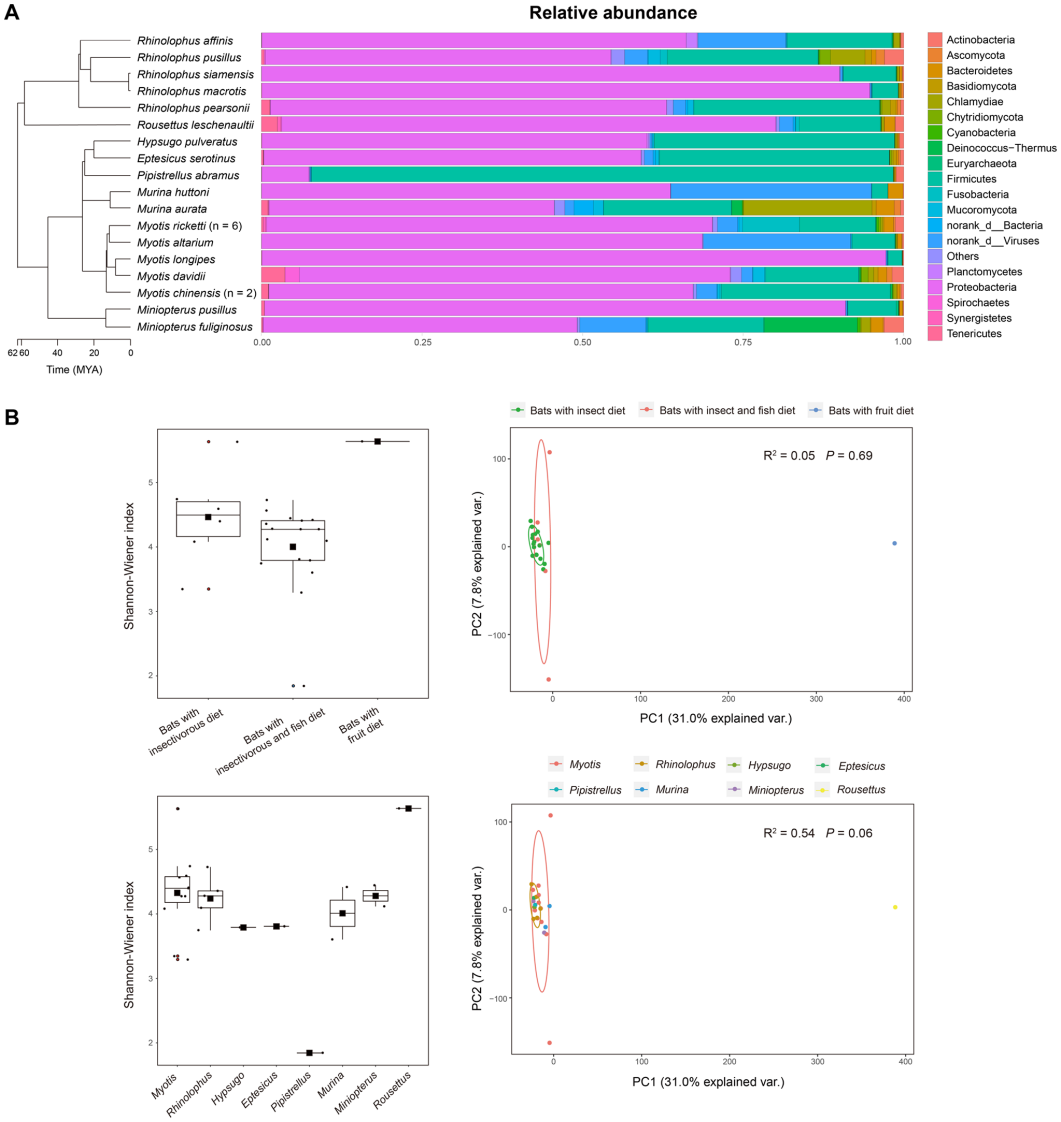
Diversity of gut microbiota in various species of bats. **(A)** Top 18 microbial phyla in gut microbiota of bats. The bar below the phylogenetic tree indicates divergence time of the bat species. **(B)** Diversity of gut microbiota in bats with different diet habits (the two upper panels) and those of different phylogenies (at genus level, the two lower panels).

After taxonomic annotation, 394 archaea species, 18,271 bacterial species, 757 fungal species, and 1,912 viral species were detected. Except for *P. abramus*, all samples showed similar microbial profiles at the phylum level (Figure 2A) with Proteobacteria being most predominant (average relative abundance: 0.658), followed by Firmicutes (0.204), norank_d__Viruses (0.054), Chlamydiae (0.018), and Deinococcus-Thermus (0.010). The predominant bacterial genera were *Enterobacter* (0.054), *Escherichia* (0.047), norank_f__Enterobacteriaceae (0.041), *Serratia* (0.040), *Klebsiella* (0.032), *Bacillus* (0.031), *Morganella* (0.030), *Lactococcus* (0.022), *Citrobacter* (0.021), *Aeromonas* (0.019), *Kluyvera* (0.017), and *Enterococcus* (0.015) (Supplementary Figure S1).

There was no significant difference in microbial diversity was detected among bats with insect (IB) or both insect and fish (IFB) diets by statistical (Wilcoxon test adjusted by the method of Holm, all *p* > 0.05) and PCA analyses (*p* = 0.69; Figure 2B). Meanwhile, in PCA analysis, there was no significant difference at the family level of microbes among bats of different phylogenies (*R*^2^ = 0.049, *p* = 0.743) (Supplementary Figure S2), but a high variation in microbial diversity at the genus level was observed (*R*^2^ = 0.54, *p* = 0.06; Figure 2B).

### Identification of microbes responsible for major variations in gut microbiota and major metabolic pathways in bats

**Figure 3:**
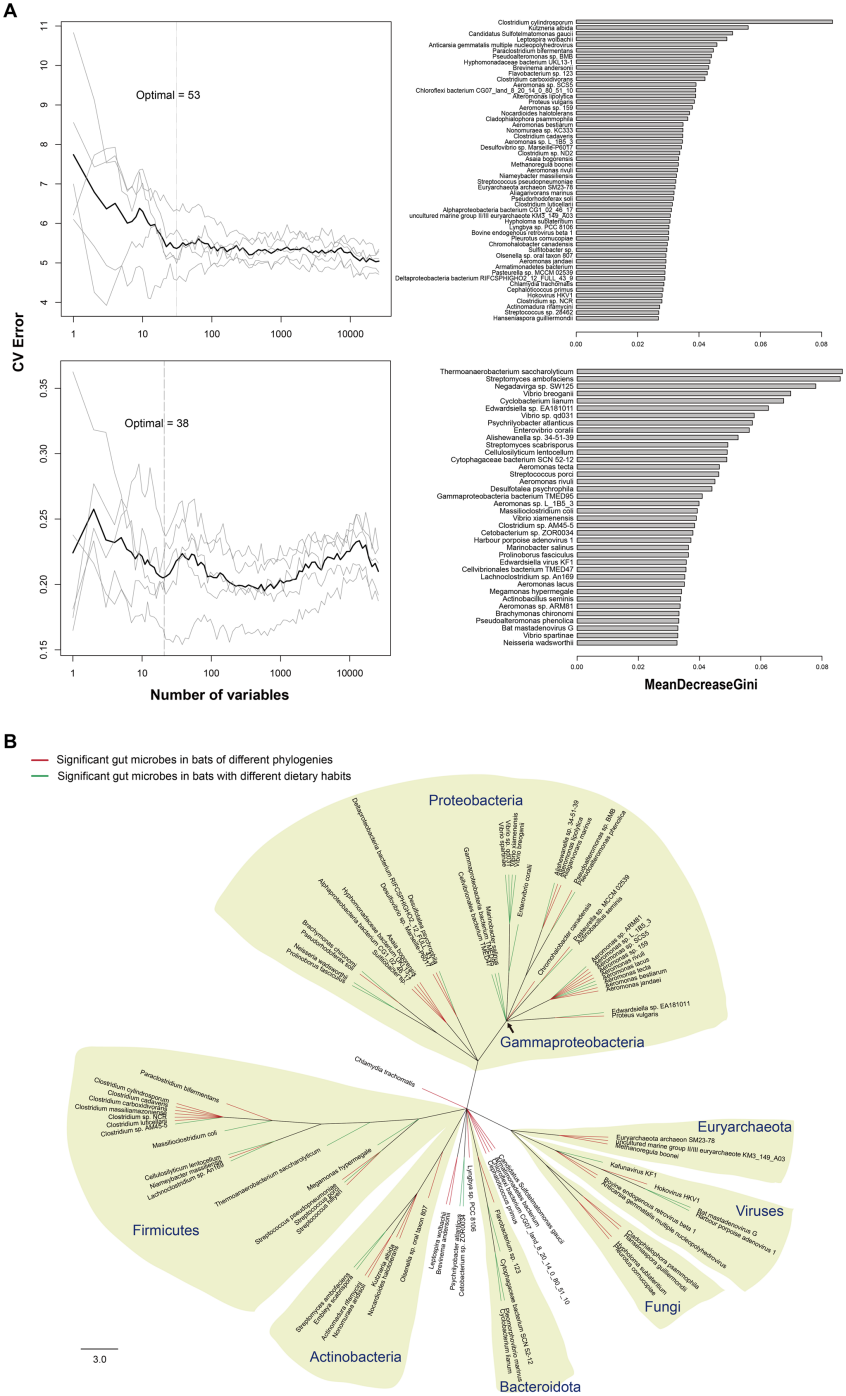
Major microbial species in gut microbiota of bats. **(A)** Gray lines show results of 5 repeats of RF modeling with 10-fold cross validations. Dark lines indicate average values of the 5 repeats. Dashed lines denote the lowest point of the dark line, where the model had the lowest out-of-bag (OOB) error. The bar length in MeanDecreaseGini shows the extent of microbes affecting node impurity in RF modeling, and a microbe with a longer bar has a higher effect on the composition of gut microbiota. **(B)** Phylogenetic tree of the 91 most significant microbes among bats. Red branches represent the 53 most significant microbes among bats of different phylogenies, and green branches denote the 38 most significant microbes among bats with different dietary habits. Scale bar length indicates the number of amino acid substitutions per site.

The random forest (RF) method was used to identify key microbes responsible for major variations in the composition of gut microbiota, and 53 different species of microbes were found among bats of different phylogenies (PG bats; OOB error rate: 41.67%). For bats with different dietary habits (DG bats), 38 different species of microbes were found (OOB error rate: 16.67%) (Figure 3A). By constructing a phylogenetic tree of all 91 microbes, species of Firmicutes, Actinobacteria, and Gammaproteobacteria were found to contribute the most variations in gut microbiota in all bats. For bats of different phylogenies, species of *Clostridium*, *Euryarchaeota*, and ancient bacterial phyla contributed the major variation. In contrast, species of *Vibrio* contributed almost solely the major variation in gut microbiota in bats with different dietary habits (Figure 3B).

For all bats, the predominant orthologous gene functions were Transcription, Replication, recombination, and repair, Amino acid transport and metabolism, and Signal transduction mechanisms (Supplementary Figure S3). The predominant metabolic pathways were Transporters for insectivorous bats (IB), Transporters and Carbon metabolism for bats that feed on both insects and fish (IFB), and Transporters and Biosynthesis of amino acids for frugivorous bat (FB) (Supplementary Figure S4). No significant difference in the number of these orthologous genes or metabolic pathways was detected among all bats. However, the number of genes of metabolic pathways was found to vary among bats with different dietary habits as follows: Photosynthesis - antenna proteins between IB and IFB (*p* = 0.036); Flavonoid biosynthesis between IB and FB (*p* = 0.0002) and between IFB and FB (*p* = 0.041); Isoflavonoid biosynthesis between IB and IFB (*p* = 0.035); Flavone and flavonol biosynthesis between IB and IFB (*p* = 0.032); and Stilbenoid, diarylheptanoid, and gingerol biosynthesis between IB and FB (*p* = 0.0002) and between IFB and FB (*p* = 0.041).

### Significantly changed microbes during dietary transition

**Figure 4:**
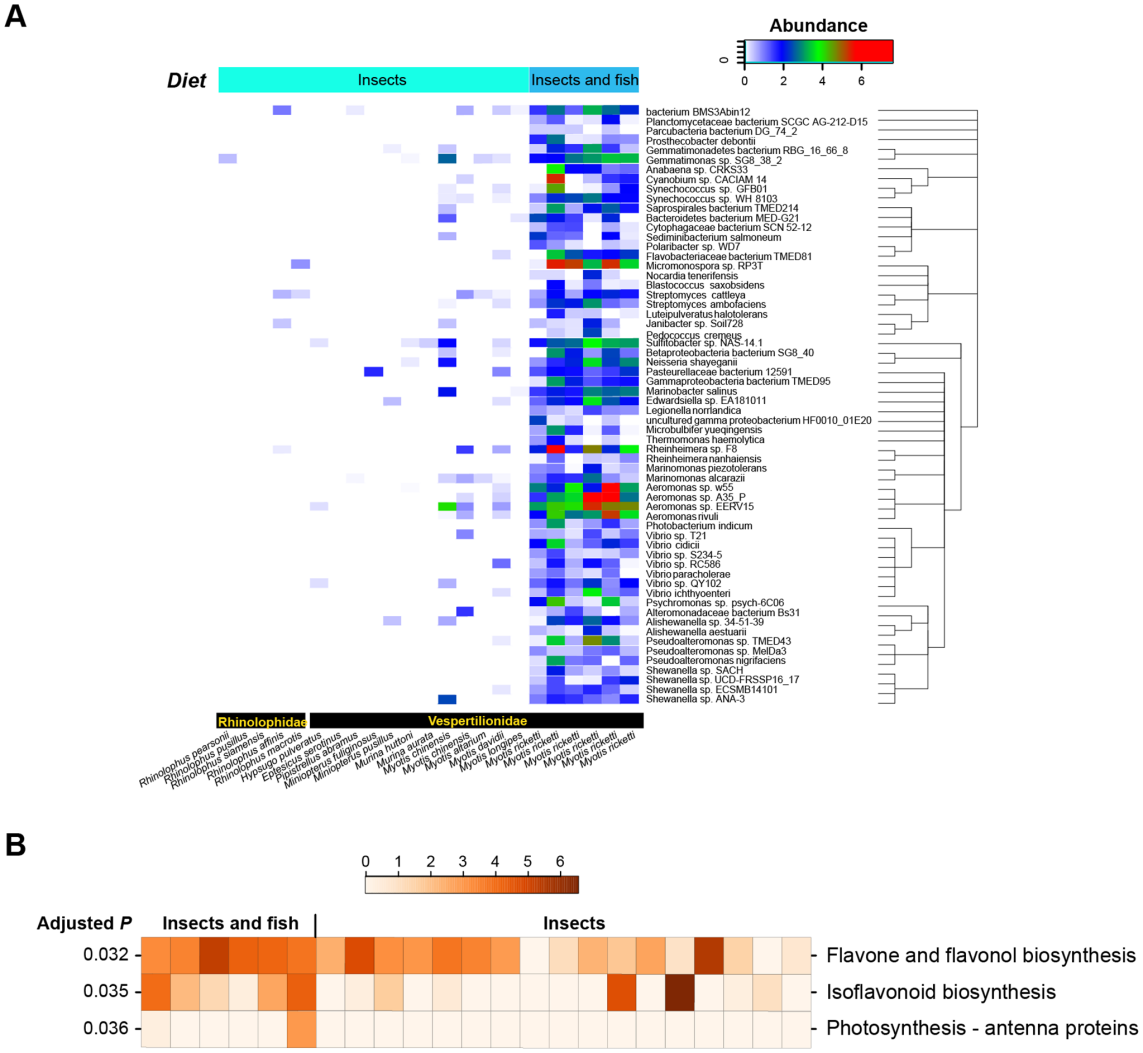
Significantly changed bacterial species and metabolic pathways between IB and IFB. **(A)** The 62 significantly changed bacterial species between IB and IFB. The phylogenetic relationships of these species are shown in the right panel. **(B)** Significantly altered KEGG pathways between IB and IFB.

By performing Metastats non-parametric multiple tests, the abundance of 62 bacterial species was found to vary (Figure 4A). Most of them were also observed in the RF modeling of bats with different dietary habits, e.g., members of *Vibrio*, *Aeromonas*, *Streptomyces*, *Cytophagaceae*, *Pseudoalteromonas*, *Alishewanella*, *Pseudoalteromonas*, *Marinobacter*, *Neisseria*, and *Gammaproteobacteria bacterium* TMED95. A large number of *Aeromonas* species were much more abundant in bats that feed on both insects and fish than in those with insect diet, and *Micromonospora* sp. RP3T was found to be enriched in bats that feed on both insects and fish (Figure 4B).

## Discussion

The compositions of gut microbiota vary among different species of animals and may also vary under different physiological and behavioral conditions. As bats are highly differentiated animals with various dietary preferences and some bat species such as *M. pilosus* are in transition from insect-eating to fish-eating, they are ideal for investigation of the effects of diets on gut microbiota. In this study, we found that the gut microbiota profile of the one frugivorous bat (*R. leschenaultii*) examined was greatly different from that of insectivorous bats and bats that feed on both insects and fish, but those of the latter two dietary types of bats were similar. However, the abundance of 62 different species of bacteria was found to vary among different species of bats. These observations suggest that the difference in the profiles of gut microbiota in bats of different phylogenies was higher than those with different dietary habits as previously described (Lutz et al., 2019). We also found a much less difference in gut microbiota among bats of different phylogenies at the genus (*R*^2^ = 0.54, *p* = 0.06) level than at the family level (*R*^2^ = 0.049, *p* = 0.743) by PCA analysis. A possible reason for such is that closely related bats have similar behaviors, physiologies, and environmental factors and therefore have similar gut microbial compositions (Archie, 2019).

Many of the physiological or behavioral characteristics of bats have a unique evolutionary history (Brualla et al., 2023). For example, the basic immune systems of bats (e.g., innate and adaptive immune systems) are more tolerant than those of other mammals (Randolph and Barreiro, 2018). A study of bat rabies virus found that bat antibodies failed to penetrate the blood-brain barrier and therefore were unable to defend the infection in the central nervous system (Roy et al., 2007). However, bats are known to carry many potentially lethal viruses, such as filoviruses, henipaviruses, lyssaviruses, paramyxoviruses, and coronaviruses (Karunarathna et al., 2020; Sun et al., 2020). Such symbiosis of various viruses may affect their physiologies, behaviors, and adaption to the virulent gut ecology. Bats also harbor many bacterial pathogens such as various species of *Bacillus*, *Enterobacter*, *Enterococcus*, *Escherichia*, *Klebsiella*, *Pantoea*, *Pseudomonas*, and *Serratia* (Daniel et al., 2013; Sun et al., 2020). We postulate that the symbiosis of various viruses and pathogenic bacteria is a survival strategy for bats to evade predators and that the host-microbe interactions may be a driving force for the evolution of bats to survive in various ecologic conditions. Dietary habits and other behaviors may also affect animal evolution. For instance, the bamboo diet promotes the adaptive development of strong teeth and paws in giant panda (Vallittu et al., 2021). Another example is that the flying ability of birds or bats is endowed with light bone, high degree of skull healing, shoulder strap, sternum with keel protuberance, and degradation of posterior tibia and fibula (Chang et al., 2019).

Different from most terrestrial mammals, the gut microbiota of bats with different dietary habits have few Bacteroidetes, but similar to carnivores, bat gut microbiota is enriched in Firmicutes and Proteobacteria (Sun et al., 2020). A previous study of bat gut bacteria showed that species of Gammaproteobacteria and Firmicutes were dominant in non-breeding adult bats; this observation is also consistent with ours in this study. We also did not observe a significant correlation between dietary habits and gut microbiota in bats that feed on insets or both insects and fish as that reported by other investigators (Phillips et al., 2012; Carrillo-Araujo et al., 2015; Lutz et al., 2019). We noticed that *Aeromonas* species were much more abundant in bats that feed on both insects and fish than those with insect diet. As *Aeromonas* is often detected in fish (Hanninen et al., 1997), it may alter the composition of gut microbiota in bats that feed on fish.

As observed in previous studies (Zepeda Mendoza et al., 2018; Ingala et al., 2021), we found that the predominant metabolic pathways were Transporters for insectivorous bats; Transporters and Carbon metabolism for bats that feed on both insects and fish; and Transporters and Biosynthesis of amino acids for frugivorous bat. A recent study showed that the numbers of genes involved in 37 metabolic pathways were different between herbivorous and animalivorous bats (Ingala et al., 2021). However, we found only 3 metabolic pathways involved in stilbenoid, diarylheptanoid and gingerol biosynthesis that were different in the number of genes between insectivorous bats and bats (*M. pilosus*) that feed on both insects and fish. A probable reason for this discrepancy is that we examined a very small dietary shift, i.e., from insect to both insect and fish diet. As *M. pilosus* bats have a high viral load, the enrichment of genes in these 3 metabolic pathways may be a symbiotic strategy with the viruses. The finding that the number of genes involved in photosynthesis of antenna proteins varied between IB and IFB (*p* = 0.036) suggests that photosynthesis also plays a role in gut microbial variation among bats with different dietary habits as previously postulated (Jin et al., 2021).

By investigating the effects of host speciation and dietary habits on gut microbiota, this study disclosed the effect of host–microbe interactions on bat evolution. A major weakness in this study is in bat sampling. To avoid the influence of geographic variation on gut microbiota, bats used in this study were captured from the same cave. As a result, we only captured bats from 3 families (Pteropodidae, Rhinolophidae, Vespertilionidae), and the number of bats is very limited with only one bat in most species. Such problem may introduce deviations in the comparison of microbial diversity. Furthermore, a previous study surveyed the variations in gut microbiota among 9 bat families, and nearly half of the families showed significant differences in gut bacterial composition (Lutz et al., 2019). We investigated 3 bat families and detected no significant difference in gut microbiota among them, indicating that investigations with more families are warranted in future studies. Although we have investigated the effects of host speciation and diets on the composition of gut microbiota, there may be other host factors that also play a role in the formation of gut microbiota in bats. We also noticed that more than 50% of gut microbial genes in each sample were annotated as “function unknown.” It has been postulated that these genes may be involved in echolocation, longevity, flying, and immune system (Zhang et al., 2013; Jebb et al., 2020).

## Conclusion

In this study, we found a very weak correlation between dietary habits and gut microbiota in bats that feed on insects or both insects and fish. However, the compositions of gut microbiota were found to vary among different species of bats, suggesting that host speciation plays a major role in the formation of gut microbiota. We also found differences in the number of genes related to certain metabolic pathways among bats with different dietary habits, suggesting the effect of diet on physiological functions of bats.

## Data availability statement

The datasets presented in this study can be found in online repositories. The names of the repository/repositories and accession number(s) can be found at: https://www.ncbi.nlm.nih.gov/, PRJNA954561.

## Ethics statement

The animal study was reviewed and approved by the Animal Ethics Committee of Institute of Zoology, Guangdong Academy of Sciences (No. GIABR20200810).

## Author contributions

MG and LZ designed the research. MG performed the research. JW, YZ, and XH collected samples and extracted fecal DNA. MG, SX, JQ, PL, CZ, and CH provided analytic tools. MG, SX, JD, and JQ analyzed data. MG and LZ wrote the paper. All authors contributed to the article and approved the submitted version.

## Funding

This work was supported by grants from the GDAS Special Project of Science and Technology Development (2021GDASYL-20210103052), the Guangdong Provincial Science and Technology Program (2021B1212110003 and 2021B1212050021), and the National Natural Science Foundation of China (32172435).

## Conflict of interest

The authors declare that the research was conducted in the absence of any commercial or financial relationships that could be construed as a potential conflict of interest.

The handling editor DJ declared a shared parent affiliation with some of the authors MG, JW, YZ, XH, PL, JD, CZ, and LZ at the time of review.

## Publisher’s note

All claims expressed in this article are solely those of the authors and do not necessarily represent those of their affiliated organizations, or those of the publisher, the editors and the reviewers. Any product that may be evaluated in this article, or claim that may be made by its manufacturer, is not guaranteed or endorsed by the publisher.

## Footnotes

^1^http://i.cs.hku.hk/~alse/hkubrg/projects/idba_ud/

^2^http://exon.gatech.edu/meta_gmhmmp.cgi

^3^http://www.bioinformatics.org/cd-hit/

^4^http://blast.ncbi.nlm.nih.gov/Blast.cgi

^5^http://www.timetree.org/
