# Supplementary figures and images for: The difference in the composition of gut microbiota is greater among bats of different phylogenies than among those with different dietary habits
Source: Front Microbiol. 2023 Jul 28;14:1207482. doi: 10.3389/fmicb.2023.1207482 (PMC10419214; doi:10.3389/fmicb.2023.1207482)

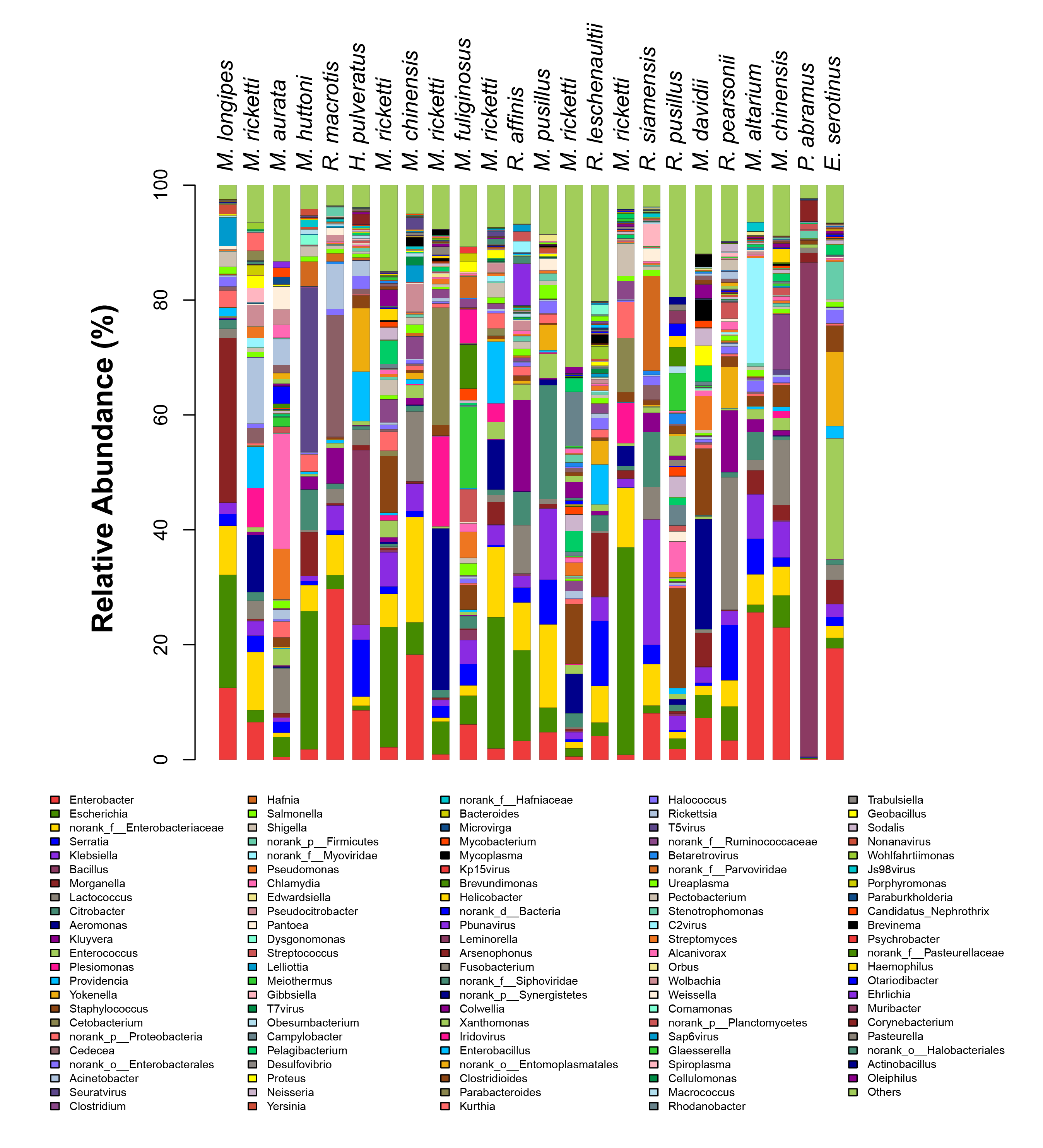

Supplement: Supplementary file 1 [file Image_1.TIF]

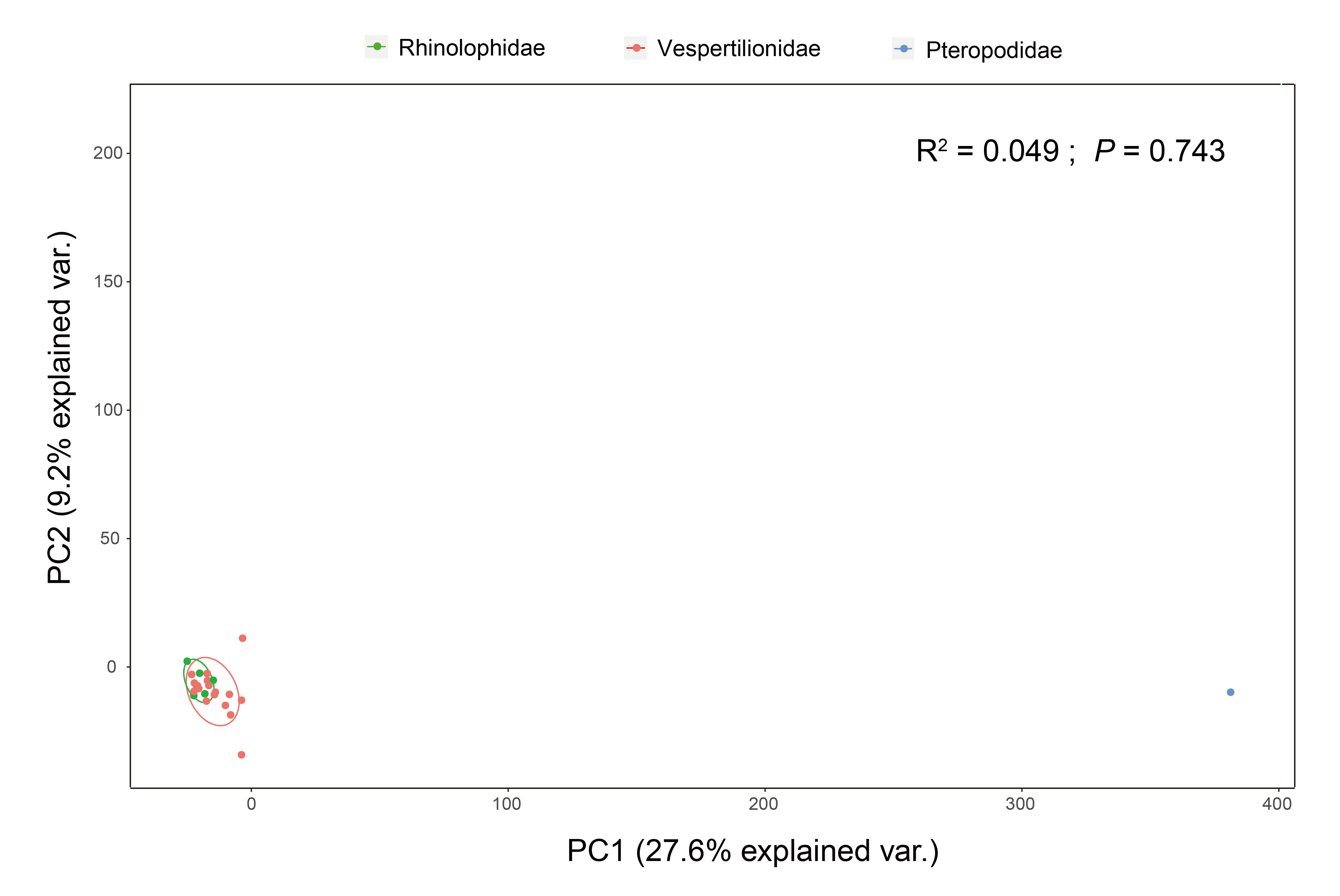

Supplement: Supplementary file 2 [file Image_2.TIF]

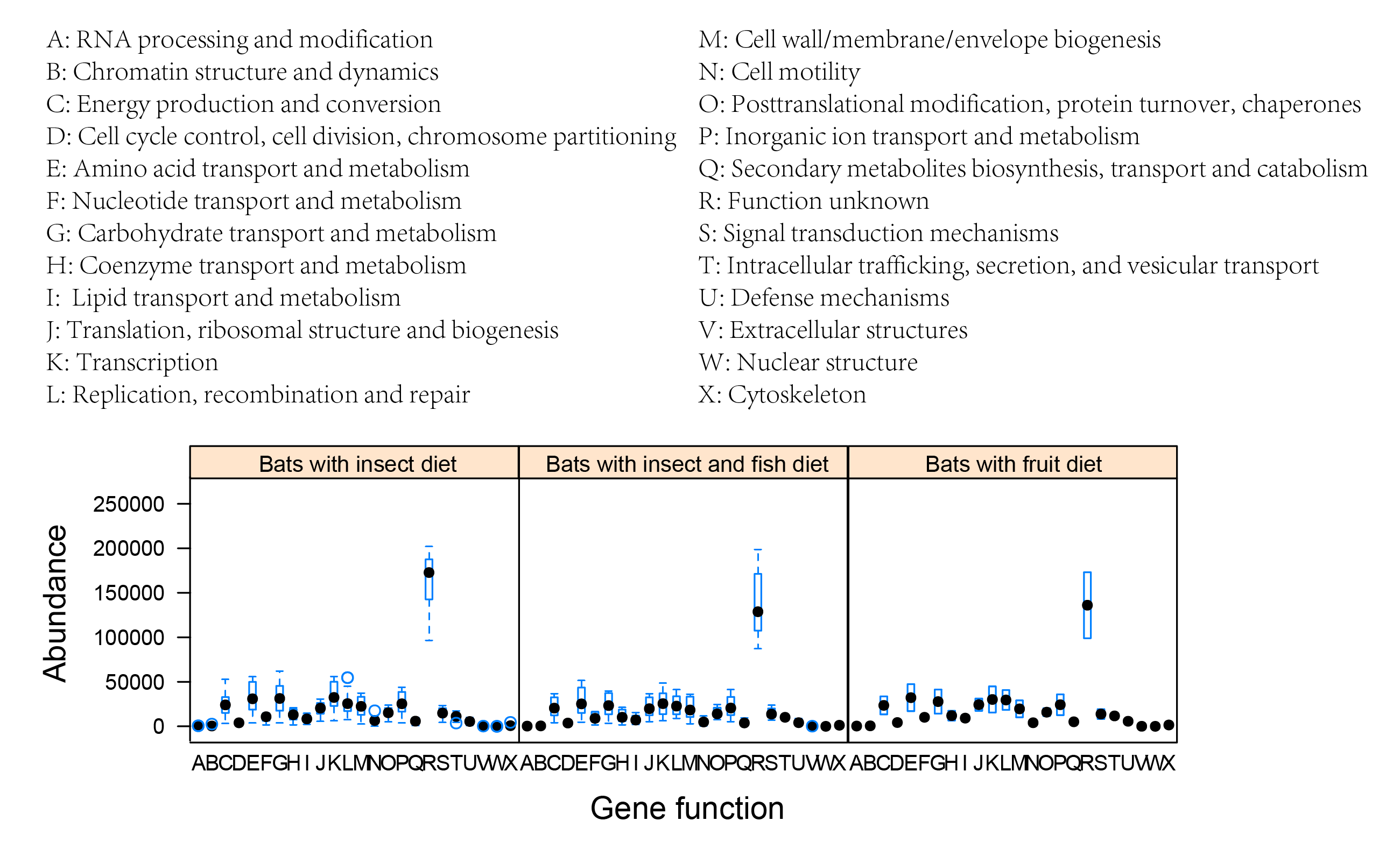

Supplement: Supplementary file 3 [file Image_3.TIF]

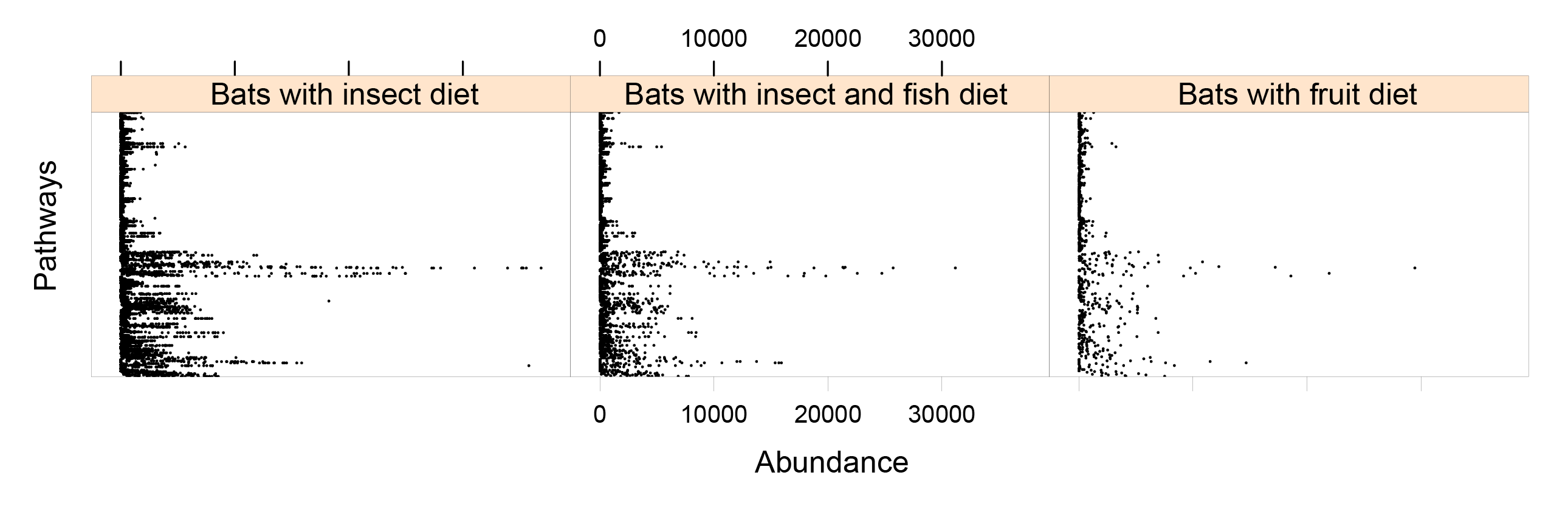

Supplement: Supplementary file 4 [file Image_4.TIF]
